# Nrf2, stress and aging

**DOI:** 10.18632/aging.102143

**Published:** 2019-08-02

**Authors:** Ioannis P. Trougakos

**Affiliations:** 1 Department of Cell Biology and Biophysics, Faculty of Biology, National and Kapodistrian University of Athens, Panepistimiopolis, Athens 15784, Greece

**Keywords:** aging, Insulin/IGF-like, metabolism, mitostasis, Nrf2, proteostasis, stress sensors

**Figure 1:**
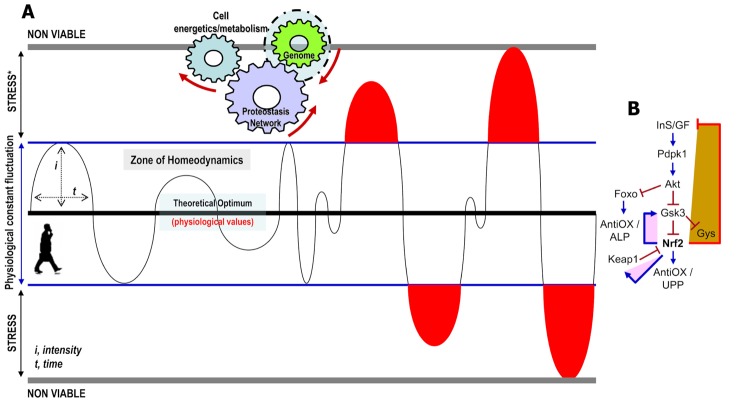
**Stress responses are normal reactions to the demands of life.** (**A**) The myriads of physicochemical parameters that characterize an organism fluctuate constantly around an optimum. The intensity and duration of fluctuation may vary for different parameters; yet by the combined action of their respective sensors (mostly transcription factors) these values tend to remain (at least while young) within a physiological range (zone of homeodynamics). The zone of stress in a medical or biological context is defined as a physical or mental condition that causes tension. Stress is caused by either molecules that exceed a physiological concentration (e.g. ROS) or by external (e.g. UV, pollutants, drugs, etc) stressors. Stress responses launch specific genomic alterations that readjust the cellular proteostatic and metabolic networks in order to normalize non-physiological values and/or neutralize external stressors. (**B**) Stress-mediated Nrf2 activation triggers (among others) an adaptive metabolic response which by suppressing (as part of a negative feedback loop) the InS/GF axis indirectly reallocates resources from growth and longevity to somatic preservation and stress tolerance [7]. In the young organism Nrf2 activation gradually relieves stress; yet, for the circuit to close this process has to be tightly linked with parallel Nrf2 inactivation. The latter is ensured by evolutionary favored build-in negative feedback loops that in the case of Nrf2 trigger both its functional inactivation (e.g. by Gsk3) and/or its physical elimination (e.g. Keap1-mediated degradation) [7]; explaining thus, why Nrf2 (and most other stress sensors) is a short-lived protein with low basal levels (→ denotes positive regulation and ┤a negative regulatory effect).

Viability of metazoans largely depends on their ability to regulate metabolic processes in order to produce energetic molecules as well as on their capacity to mount anti-stress responses [1]. These processes are regulated in real-time by a network of sensors (mostly transcription factors) which monitor organismal physicochemical parameters and constantly trigger genomic responses aiming to restore optimal (evolutionary set) values and normal cellular functionality (Figure 1A). At the whole organism level, these responses require complex co-regulation and wiring of cell-autonomous and non-autonomous mechanisms [2]; which however, decline during aging leading to increased morbidity and mortality [3]. The network of cellular sensors comprises numerous short-lived proteins, including nuclear factor erythroid 2 like 2 (NFE2L2/Nrf2) which reportedly modulates cell responses against oxidative/xenobiotic damage [4]. Nrf2 is subject to inactivation by several kinases including Glycogen synthase kinase 3β (Gsk3) and tyrosine kinase Fyn, as well as to Keap1-mediated proteasomal degradation [1,4].

Recent work from our lab in the fly model showed that increased proteome instability due to proteasome dysfunction [5] or disruption of mitochondrial functionality [6] activated cap-n-collar isoform-C (the Nrf2 ortholog in *Drosophila* that combines the functions of the mammalian Nrf1 and Nrf2 genes [4]) to upregulate cytoprotective antioxidant, proteostatic and mitostatic modules; consistently, Nrf2 overexpression in flies conferred stress tolerance [7]. Yet, while mild Nrf2 activation extended lifespan, sustained Nrf2 overactivation resulted in larval lethality and if induced in adult flies it sharply reduced longevity [7]. Thus, paradoxically enough, Nrf2 overactivation reduces lifespan while at the same time the organism is in a state of maximum stress tolerance, indicating that the activation level of Nrf2 that enhances healthspan/lifespan is considerably lower than that which maximizes cytoprotection. Further studies also revealed that Nrf2 modulates basal mitochondrial functionality and that prolonged Nrf2 overactivation reprogrammed cellular bioenergetics resulting in the appearance of diabetic phenotypes [7]; therefore, Nrf2 is far more than stress neutralizer. Mechanistically, the diabetic phenotype is caused due to Nrf2-mediated (as part of a negative feedback loop) suppression of the Insulin/IGF-like (InS/GF) signaling (Figure 1B). Interestingly, Nrf2 apart from Keap1 also upregulated its other inhibitor, namely Gsk3 (a target for negative regulation by InS/GF), indicating that as the Nrf2 network (and likely of all other sensors) evolved in higher metazoans one major adaptation was the limitation of its own activity. Prolonged Nrf2 overactivation also suppressed the expression of proteins involved in flies’ courtship behavior, mating and reproduction, sleep and circadian rhythms, indicating that aberrant activation of stress sensors (e.g. Nrf2) affects numerous regulatory networks of metazoans. Similar effects were noted after muscle-targeted Nrf2 overactivation suggesting the existence of a dynamic communication between stress pathways in muscle and adaptive programs in other peripheral organs that are activated through central integration of signals spanning multiple tissues. Early genetic or dietary suppression of the InS/GF axis titrated the Nrf2 transcriptional activity to lower levels (e.g. due to Gsk3 activation) (Figure 1B) and extended the Nrf2 overexpressing flies’ lifespan. Thus, suppression of the InS/GF axis is dominant over stress indicating possible therapeutic dietary interventions for various age-related diseases of chronic stress.

Taken together, these findings suggest that even in the absence of damage, persistent stress signaling triggers an adaptive metabolic response which reallocates resources from growth and longevity to somatic preservation and stress tolerance. This notion provides a reasonable explanation of why most cytoprotective stress sensors are short-lived proteins, and it also explains the build-in negative feedback loops; the low basal levels of these proteins, and why their suppressors were favored by evolution. Nonetheless, none of the severe adverse effects induced by Nrf2 overactivation is sufficient reason to discredit the Nrf2 pathway as a drug target, e.g., for anti-aging purposes. Evidence comes from the increased flies’ healthspan associated with mild Nrf2 activation, and also from the fact that humans have been safely ingesting Nrf2 activators in their diet for millennia; to this end, the druggable Gsk3 [8] and Fyn kinases are promising candidates for the identification of novel Nrf2 activators. Furthermore, a detailed understanding of the correct time (*when*), dose (*how much*) or tissue-targeted (*where*) interventions with stress sensors activators and of their interactions with disease-related pathways remains critical to avoid clinical trial failures. Additional topics to be addressed include a distinction between true stress and normal fluctuations in the zone of homeodynamics; whether the critical determinant of stress is the type, the level or the primary site at which it occurs; which are the age-dependent changes in sensors functionality, and if there are sensors more vulnerable to the aging process; and finally, whether a “loss” of a sensor can be compensated for by the remaining ones. Systematic analyses of these questions in model organisms can provide valuable preclinical insights and elucidate potential therapeutic avenues against aging and/or age-associated pathologies.
